# Supplementary material for: Gemin4 is an essential gene in mice, and its overexpression in human cells causes relocalization of the SMN complex to the nucleoplasm
Source: Biol Open. 2018 Jan 25;7(2):bio032409. doi: 10.1242/bio.032409 (PMC5861365; doi:10.1242/bio.032409)
Supplement: Supplementary information [file biolopen-7-032409-s1.pdf]

Supplemental Figure

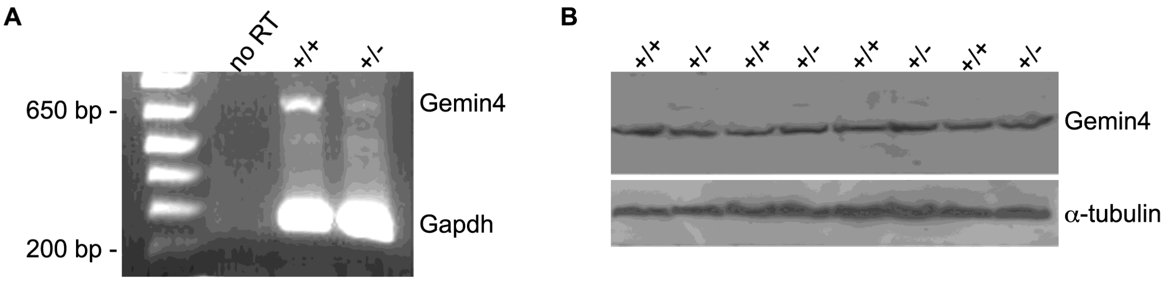

Figure S1. Expression of Gemin4 mRNA and protein. (A) RNA was isolated from livers of P4 *Gemin4*$^{+/-}$ and *Gemin4*$^{+/+}$ mice and semi-quantitative RT-PCR analysis was performed. Pooled *Gemin4*$^{+/-}$ and *Gemin4*$^{+/+}$ RNA for no RT control. (B) Western analysis of liver lysates was performed to determine protein levels of Gemin4 in wild-type and heterozygous animals; α-tubulin was used as a loading control.

Biology Open • Supplementary information
